# First experimental evidence suggests use of glucobrassicin as source of auxin in drought-stressed *Arabidopsis thaliana*

**DOI:** 10.3389/fpls.2022.1025969

**Published:** 2022-10-31

**Authors:** Johann Hornbacher, Ina Horst-Niessen, Cornelia Herrfurth, Ivo Feussner, Jutta Papenbrock

**Affiliations:** ^1^ Institute of Botany, Leibniz University Hannover, Hannover, Germany; ^2^ Albrecht-von-Haller-Institute for Plant Sciences, Department of Plant Biochemistry, University of Göttingen, Göttingen, Germany; ^3^ Göttingen Center for Molecular Biosciences (GZMB), Service Unit for Metabolomics and Lipidomics, University of Göttingen, Göttingen, Germany; ^4^ Göttingen Center for Molecular Biosciences (GZMB), Department of Plant Biochemistry, University of Göttingen, Göttingen, Germany

**Keywords:** drought stress, glucobrassicin (PubChem CID: 5484743), glucosinolates, Indole - 3 - acetic acid (IAA), turnover (TO), auxin

## Abstract

The synthesis of indole-3-acetonitrile (IAN) from the indolic glucosinolate (iGSL) glucobrassicin (GB) is a unique trait of members of the Brassicales. To assess the contribution of this pathway to indole-3-acetic acid (IAA) synthesis under stress conditions, drought stress (DS) experiments with *Arabidopsis thaliana* were performed *in vitro*. Analysis of GSLs in DS plants revealed higher contents of GB in shoots and roots compared to control plants. Deuterium incorporation experiments showed the highest turnover of GB compared to all other GSLs during drought conditions. Evidence suggests the involvement of the thioglucosidase BGLU18 in the degradation of GB. The nitrile specifier proteins NSP1 and NSP5 are known to direct the GSL hydrolysis towards formation of IAN. Nitrilases like NIT2 are able to subsequently synthesize IAA from IAN. Expression of *BGLU18, NSP1*, *NSP5* and *NIT2* and contents of GB, IAN and IAA were significantly elevated in DS plants compared to control plants suggesting the increased use of GB as IAA source. Significantly higher contents of reactive oxygen species in DS *bglu18* and *epithionitrile specifier protein* (*esp)* mutants compared to Col-0 indicate higher stress levels in these mutants highlighting the need for both proteins in DS plants. Furthermore, GB accumulation in leaves was higher in both mutants during DS when compared to Col-0 indicating enhanced synthesis of GB due to a lack of breakdown products. This work provides the first evidence for the breakdown of iGSLs to IAN which seems to be used for synthesis of IAA in DS *A. thaliana* plants.

## 1 Introduction

In the past, research mainly focused on anticipatory, repellant or toxic effects of glucosinolates (GSLs) or their breakdown products. The actions against biotic stressors and subsequent change in GSL contents is well described (Kliebenstein et al., 2005; Wittstock and Burow, 2010). Only recently, data was published indicating that GSLs exhibit functions in abiotic as well as biotic stress situations. It was found that isothiocyanates (ITCs) derived from aliphatic GSLs (aGSLs) are involved in stomatal closure in *Arabidopsis thaliana* and are therefore major contributors in the regulation of water homeostasis of plants (Khokon et al., 2011). However, no published data is available about the role of indolic GSLs (iGSLs) in abiotically stressed plants.

Drought is one of the major reasons for crop losses worldwide (Matiu et al., 2017). Many crop plants grown worldwide belong to the Brassicaceae family. Among them are crops grown for human nutrition like cabbage (*Brassica oleracea* var. *capitata*) and broccoli (*Brassica oleraceae* var. *italica*). Members of this family like canola (*Brassica napus*) and *Crambe abyssinica* are also grown for industrial purposes showing the widespread use of this family in agriculture (Warwick, 2011; Zorn et al., 2019). Investigation of the behavior of specialized metabolites synthesized by the Brassicaceae in response to drought stress could be beneficial when it comes to the selection of drought tolerant varieties.

**Figure 1:**
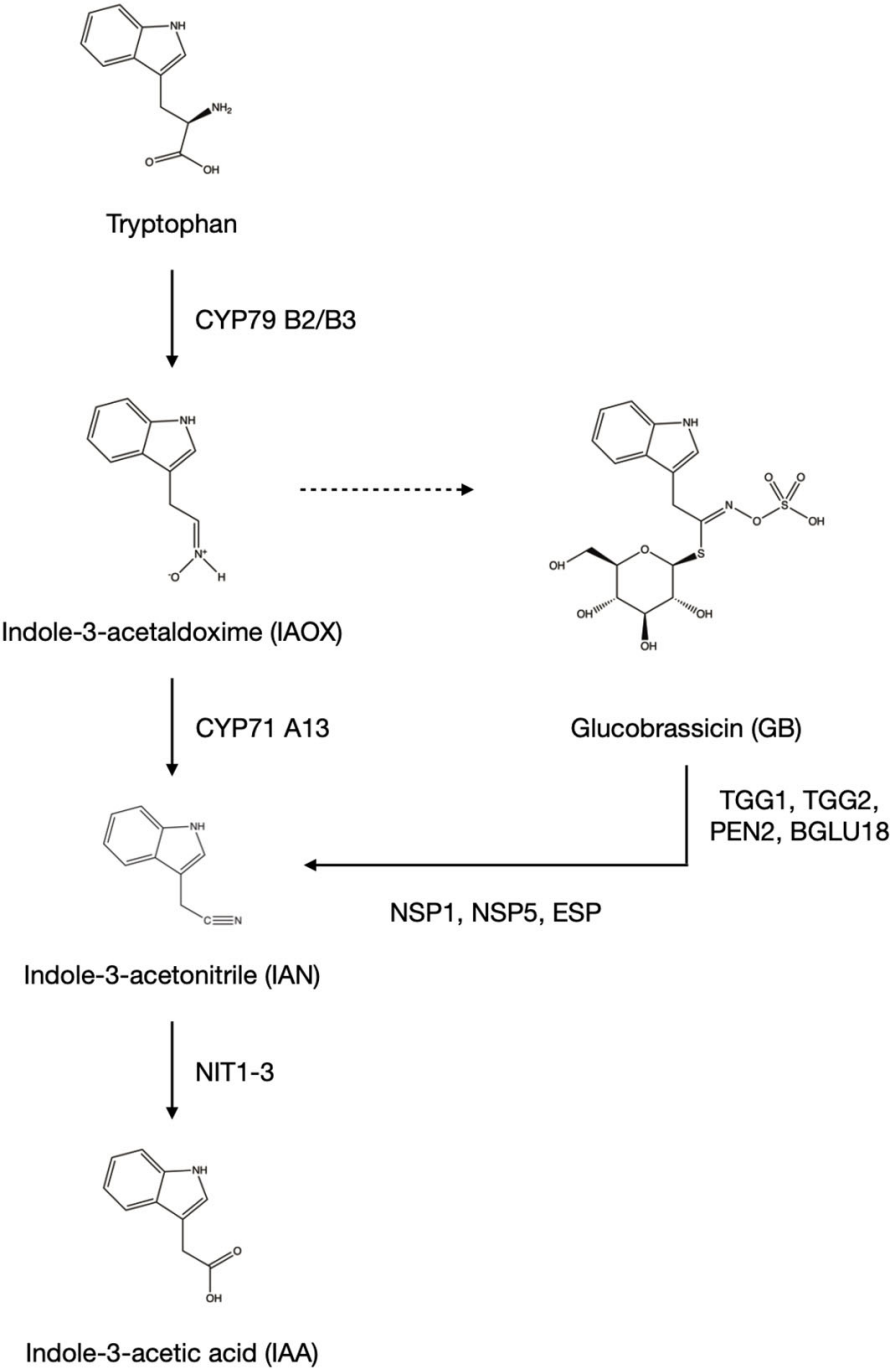
Synthesis of indole-3-acetic acid (IAA) in Arabidopsis thaliana. Members of the Brassicaceae family are able to synthesize IAA through additional synthetic pathways starting from indole-3-acetaldoxime (IAOX). IAA is synthesized from the common intermediate indole-3-acetonitrile (IAN), either coming directly from indole-3-acetaldoxime (IAOX), or the breakdown of the indolic glucosinolate glucobrassicin (GB). The dotted line from IAOX to GB indicates multiple steps. The figure was created using ChemDraw Professional Version 20.0.0.38 (PerkinElmer, Waltham, United States).

Glucosinolates are specialized metabolites synthesized by members of the Brassicales order. Dependent on the amino acid they are derived from, they are subdivided into aliphatic GSLs aGSLs derived from alanine, valine, leucine, isoleucine and methionine, and indolic GSLs iGSLs derived from tryptophan. The synthesis of indole-3-acetalaldoxime (IAOX) from tryptophan performed by CYP79B2/B3 (**Figure 1**) is limited to members of the Brassicaceae. This intermediate gives rise to either indole-3-acetic acid (IAA) through the intermediate indole-3-acetonitrile (IAN) with the action of CYP71A13 or iGSLs (Bekaert et al., 2012).

Glucosinolates and classical thioglucosidases (EC 3.2.1.147) are either stored in separate cells or cell compartments. Thioglucosidases are subdivided into classical (e.g. TGG1, TGG2) and atypical groups (e.g. PEN2, BGLU18). In the active site of the classical thioglucosidases, a Glu residue is crucial for the nucleophilic attack, while a Gln residue is involved in the hydrolysis in the presence of ascorbic acid and water (Wittstock & Burow, 2010). Atypical thioglucosidases on the other hand, perform an acid/base catalysis with two Glu residues at their catalytic site (Chhajed et al., 2019). Both GSLs and thioglucosidases can come into contact if tissue disruption, e.g. through a herbivoric attack, occurs. However, turnover of GSLs also takes place in intact tissues during different developmental stages, or sulfur and nitrogen shortage (Jeschke et al., 2019). It is hypothesized that atypical thioglucosidases are most likely involved in the GSL turnover in intact tissue, because GSL contents were unaffected by the lack TGG1 and TGG2 in germinating *A. thaliana* (Meier et al., 2019). Additionally, the atypical thioglucosidase BGLU18 was reported to be localized in endoplasmic reticulum (ER)-bodies and therefore can be localized in the same cells, though in different organelles, as GSLs (Han et al., 2020).

Once GSLs and thioglucosidases come in contact, an unstable aglucone is formed, quickly reacting to isothiocyanates, thiocyanates, nitriles and epithionitriles depending on the pH, presence of ions and specifier proteins. If specifier proteins are not present, isothiocyanates (ITC) are formed, which can be conjugated to glutathione and further converted to amines and raphanusamic acid (RA) (Jeschke et al., 2019). It has be shown that RA exhibited growth inhibitory actions in Brassicaceae and non-Brassicaceae alike (Inamori et al., 1992). If nitrile specifier proteins (NSP) 1 and 5 or the epithionitrile specifier protein (ESP) are present, the outcome of the reaction is shifted towards generation of nitriles rather than isothiocyanates (Burow et al., 2008; Wittstock et al., 2016).

Degradation of the iGSL glucobrassicin (GB) in the presence of NSP or ESP yields IAN which can be converted to the auxin indole-3-acetic acid (IAA) enhancing the plants biosynthetic options by one further pathway (**Figure 1**). The most abundant auxin IAA can be synthesized through the indole-3-pyruvic acid pathway common to all plant species. In addition to that, members of the Brassicaceae are able to use IAOX, which is synthesized from tryptophan by CYP79B2/B3, as intermediate for the synthesis of IAA (**Figure 1**). The IAA precursor IAN is either synthesized directly from IAOX by CYP71A13, or by the synthesis of the iGSL GB and its subsequent breakdown (Malka & Cheng, 2017).

If specifier proteins are not present during the breakdown of GB, an array of breakdown products is produced including indole-3-carbinol (I3C) which readily forms adducts with ascorbic acid (indole-3-methyl-ascorbate or ascorbigen), cysteine (indole-3-methyl-cysteine) and glutathione (indole-3-methyl-glutathione) (Kim et al., 2008). Modelling experiments revealed docking of I3C-derived breakdown products to the auxin receptor transport inhibitor response 1 (TIR1) and hindering formation of the TIR1/IAA complex resulting in auxin antagonistic effects. For some of these products, the calculated dissociation constant was even lower compared to IAA, suggesting a tighter fit of the TIR1 complex and therefore higher antagonistic effects compared to compounds with a looser fit. It was hypothesized, that the GB breakdown regulation can be seen as a molecular switch bringing another possibility to control auxin signaling to the table (Vik et al., 2018).

The effect of water stress on GSL contents was previously observed in *A. thaliana* indicating that behavior of GSLs depends on duration and strength of the applied drought stress. However, either the publication focused on aGSLs, because contents of iGSL were unaltered by drought stress (Salehin et al., 2019), or an increase in aGSLs was only observed after additional feeding experiments with *Brevicoryne brassicae* (Khan et al., 2010).

It was hypothesized, that IAA can be formed by the breakdown of GB by piecing together different parts of the pathway (Malka & Cheng, 2017). However, no coherent data was published so far analyzing the contents of indolic metabolites and the transcription of enzymes involved in this pathway and therefore a connection between GB and IAA was never shown.

Since higher auxin contents were found to be beneficial to drought-stressed *A. thaliana*, the question arises if GB is used as a considerable source for IAA synthesis (Shi et al., 2014). To answer this question, Col-0 as well as mutants lacking genes in several key IAA synthesis steps were analyzed in control and drought conditions in this study. Analysis of GSL contents and expression analysis give first insights into the contribution of iGSLs to the synthesis of IAA in stress conditions.

## 2 Experimental procedures

### 2.1 Plant cultivation

#### 2.1.1 Experiments performed on soil

Seeds were sown on soil (Einheitserde, Sinntal-Altengronau, Germany) and transferred into pots with a diameter of 6 cm one week after germination. Pots were filled uniformly with the same amount of soil by weighing the pots. Samples of the soil used were taken and dried for 24 h at 70°C to determine the dry weight of the soil. Plants were grown for five weeks prior to stress application with a 10 h light/14 h dark cycle at 120 µmol m^2^ s^-1^ with a temperature of 21°C at daytime and 18°C at nighttime.

Drought stress was applied by desiccation of pots until the desired water content of 40% w/w was reached and holding that water content for five days by checking weight of the pots and watering if needed with deionized water. Drought stress was applied by holding the water content of the pots at 40% for five days. Plants were harvested in triplicate consisting of three pooled plants on the 5th day after starting withholding water. Drought stress on soil was applied three times with the same outcome. Results of one representative experiment consisting of three biological replicates with three pooled plants each is presented in this study.

### *2.2 In vitro* experiments

Plants were grown on petri dishes (**Supplementary Figure 2G–J**) containing 25 ml of half strength Murashige & Skoog medium and vitamin mixture solidified with 8 g L^-1^ agarose (Duchefa, Haarlem, Netherlands). Four round disks with a total weight of 5 g were removed from the petri dishes. Seeds were sterilized with 70% ethanol for 5 min, followed by incubation with 6% sodium hypochlorite (Roth, Karlsruhe, Germany) for 10 min under continuous agitation. Seeds were washed five times with sterile ultrapure water. Two seeds were placed on the petri dishes equidistant from two removed disks and the edge of the dish to obtain eight seeds in total per dish. Petri dishes were sealed with micropore tape (3M, Neuss, Germany). After one week of germination, spare seedlings were removed until four seedlings were left. Plants were grown for five weeks prior to stress application with a 10 h light/14 h dark cycle at 120 µmol m^2^ s^-1^ with a temperature of 21°C at daytime and 18°C at nighttime. Drought stress was applied by supplying the petri dishes with 5 ml of either 20% or 40% polyethylene glycol (PEG) 20,000 (Sigma-Aldrich, Taufkirchen, Germany) in the previously prepared holes of the agarose for 7 days. Plants subjected to 20% PEG were considered to be mildly drought-stressed (MDS) while plants subjected to 40% PEG were considered to be severely drought-stressed (SDS). After 7 days of drought stress, rosettes and roots of plants were harvested separately and immediately frozen in liquid nitrogen.

One exemplary experiment consisting of three biological replicates consisting of four pooled plants each is presented in this study.

Experiments analyzing SDS plants were performed twice independently *in vitro*. Due to slightly differing overall GSL contents and transcription levels, calculating the mean of the two experiments was refrained from. Instead, all data of the second repetition of the SDS experiment is shown in the supplemental part of this publication.

### 2.3 Stress status of plants

Water content of leaves was calculated by weighing frozen fresh leaf samples, lyophilization, weighing the dry weight and calculating evaporated water content.

Reactive oxygen species (ROS) were analyzed with a method developed on the basis of the oxygen radical absorbance capacity (ORAC) assay (Huang et al., 2002; Gillespie et al., 2007). Extraction of plant material was performed according to Boestfleisch et al. (2014). Extracts and a 96-well microplate (Greiner Bio-One, Frickenhausen, Germany) were kept on ice and 20 µl of 1:100 diluted extracts and 20 µl of standards, followed by 80 µl 75 mM phosphate buffer (pH 7.4) were transferred to the plate. A serial dilution (11-0.17 mM) of the standard 2,2′-azobis(2-amidino-propane) (AAPH) (Sigma-Aldrich) was prepared using phosphate buffer. Finally, 120 µl 112 nM fluorescin (Sigma-Aldrich) in phosphate buffer was added to the plate. The plate was incubated at 37°C and fluorescence was analyzed after 20 min at 485/520 nm. Destruction of fluorescin by ROS was calculated using the AAPH standard curve and contents are expressed as AAPH equivalents (AAPHE).

### 2.4 Glucosinolate analysis

Extraction and analysis of GSLs was performed according to Hornbacher et al. (2019). Glucosinolates were identified according to their specific mass fragments: glucoiberin (685, 378, 343), glucoraphanin (713, 392, 357), glucoalyssin (741, 406, 371), glucoerucin (681, 376, 341), glucohirsutin (825, 448, 413), glucobrassicin (735, 403, 368), 4-methoxyglucobrassicin (795, 433, 398) and neoglucobrassicin (795, 433, 398). Because of the exact same molar mass, identity of the two GSLs 4-methoxyglucobrassicin and neoglucobrassicin was assured with GSL analysis of *cyp81F4* which is lacking neoglucobrassicin, but not 4-methoxyglucobrassicin (Kai et al., 2011).

### 2.5 Analysis of D_2_O incorporation into glucosinolates

Plants were grown *in vitro* exactly as stated above. At the beginning of the 7-day-long drought stress period, plants were either subjected to 30% D_2_O additionally to 40% PEG 20,000 or 30% D_2_O alone. Incorporation of deuterium into GSLs was analyzed by calculating the monoisotopic and isotopomeric percentage of the total GSL content using mass chromatograms. One incorporation experiment consisting of three biological replicates made up of four pooled plants each is presented in this study

### 2.6 Transcription analysis

RNA isolation and reverse transcription were performed as described by Horst et al. (2009) with modifications. Integrity of isolated RNA was checked by gel electrophoreses. Yield of isolated RNA was between 60-120µg/µl with a ratio of 260/280 between 1.8 and 2.0.

To remove DNA, 1.2 units of DNaseI (ThermoFisher, Dreieich, Germany) per 250 ng of RNA were added and reactions were incubated for 30 min at 37°C, followed by a denaturation step of 15 min at 70°C. Synthesis of cDNA was performed with approximately 250 ng of total RNA, 50 pmol of random nonamer primer (5’NNNNNNNNN3’) and 10 pmol oligo-dT primer (5’ TTTTTTTTTTTTTTTTTT 3’). Reactions were incubated for 5 min at 70°C and cooled down on ice before adding 200 units of Moloney murine leukemia virus reverse transcriptase (Promega, Walldorf, Deutschland) and 1 mM deoxyribonucleotide triphosphates in reaction buffer as specified by the manufacturer. mRNA was amplified from cDNA using primer systems (**Supplementary Table S1**). All primer systems are located in between one single exon to ensure same product size of DNA standards as well as RNA obtained cDNA templates. Efficiency of DNA digestion was controlled by reactions without reverse transcriptase. To test linearity of cDNA synthesis at least one RNA sample of each extraction was diluted 1:4 and 1:16.

Desalted oligonucleotides were ordered from Eurofins Genomics Germany GmbH. Specificity of primer systems were positively checked *in silico* by Primer-BLAST (Ye et al., 2012), by agarose gel electrophoreses and melting curves. All primer systems are located in between one single exon to ensure same product size of DNA standards as well as RNA obtained cDNA templates. Standard curves were used in every qPCR run. Primer systems were designed in a way that all possible splice variants are measured. See **Supplementary Table S1** for primers used.

Quantitative PCR was performed on StepOne™ Plus (Applied Biosystems, Waltham, United States) with fast cycling mode (50°C 2 min, 95°C 2 min, 40 cycles of 95°C 3 sec and 60°C 30sec) using SYBR Green fluorescence (PowerUp™ SYBR™ GreenMaster Mix, ThermoFisher, Dreieich, Germany) for detection. The template concentration was 1/10 of 10 µl total volume. Oligonucleotide concentration was 300 nM each. Melting curve was preformed from 60 to 95°C in 0,3°C steps. Data analysis was done by StepOne™Software Version 2.3. The no template control always showed no amplification. Quantification of samples was done in in the range of the standard curve. Expression is presented relative to the reference gene *EF1α*.

### 2.7 Analysis of raphanusamic acid, indole-3-acetonitrile and indole-3-acetic acid

Metabolites were extracted with methyl-*tert*-butyl ether (MTBE), reversed phase-separated using an ACQUITY UPLC^®^ system (Waters Corp., Milford, MA, USA) and analysed by nanoelectrospray ionization (nanoESI) (TriVersa Nanomate^®^; Advion BioSciences, Ithaca, NY, USA) coupled with an AB Sciex 4000 QTRAP^®^ tandem mass spectrometer (AB Sciex, Framingham, MA, USA) employed in scheduled multiple reaction monitoring mode according to Herrfurth & Feussner (2020). The reversed phase separation of constituents was achieved by UPLC using an ACQUITY UPLC^®^ HSS T3 column (100 mm x 1 mm, 1.8 μm; Waters Corp., Milford, MA, USA). Solvent A and B were water and acetonitrile/water (90:10, v/v), respectively, both containing 0.3 mmol/l NH_4_HCOO (adjusted to pH 3.5 with formic acid).

For absolute quantification of raphanusamic acid, indole-3-acetonitrile and indole-3-acetic acid, 50 ng 2-oxothiazolidine-4-carboxylic acid (Merck KGaA, Darmstadt, Germany) and 20 ng D_5_-indole-3-acetic acid (Eurisotop, Freising, Germany) were added to the plant material before extraction. After extraction, the polar and non-polar phases were combined before drying under streaming nitrogen. Mass transitions and optimized parameters for the detection of these compounds are shown in **Supplemental 16**.

### 2.8 Statistical analysis

All statistical analyses were performed using InfoStat Version 2012 (University of Córdoba, Argentina). Analysis of variance (ANOVA) was performed and significant differences (p<0.05) were determined using Tukey’s test.

## 3 Results

### 3.1 Mildly and severely drought-stressed plants showed physiological differences

*Arabidopsis thaliana* Col-0 plants were subjected to different concentrations of PEG 20,000 to establish mild (MDS) and severe drought stress (SDS) conditions. To ensure reliable differences between treatments, leaf water content, oxidative stress, expression of drought-induced genes and phenotypical analyses were performed.

Leaf water content was significantly lower in MDS and SDS Col-0 plants compared to control plants (**Supplementary Figures 2A, B**). The difference in water content of 7% between control and SDS plants (**Supplementary Figure 2B**, **Supplementary Figure 3A**) was much greater compared to MDS with a difference of 2% (**Supplementary Figure 2A**). The amount of ROS was significantly higher in MDS and SDS plants compared to control plants and was overall similar in MDS and SDS plants (**Supplementary Figures 2C, D**, **Supplementary Figure 3B**). Expression of *P5CS1* was higher in SDS and MDS compared to control plants, but was higher in SDS when compared to MDS plants (**Supplementary Figures 2E, F**, **Supplementary Figure 3C**). Overall appearance of plants that were subjected to MDS (**Supplementary Figure 2H**) were visually not different from control plants (**Supplementary Figure 2G**), whereas SDS plants were smaller in size, and younger leaves were darker in color (**Supplementary Figure 2J**) compared to control plants (**Supplementary Figure 2I**). No signs of senescence or chlorosis indicating severe irreparable damage to the plants were observed in MDS or SDS plants (**Supplementary Figures 2H, J**).

Overall, MDS and SDS plants differed significantly from controls in all analyzed parameters. Differences were also observed between MDS and SDS plants in leaf water content, expression of *P5CS1* and the phenotypical analysis.

### 3.2 Glucosinolate contents differed between mildly and severely drought-stressed plants

Single GSL contents were analyzed in leaves and roots of control and DS plants to gain insight into the effects of drought on GSL metabolism.

**Figure 2:**
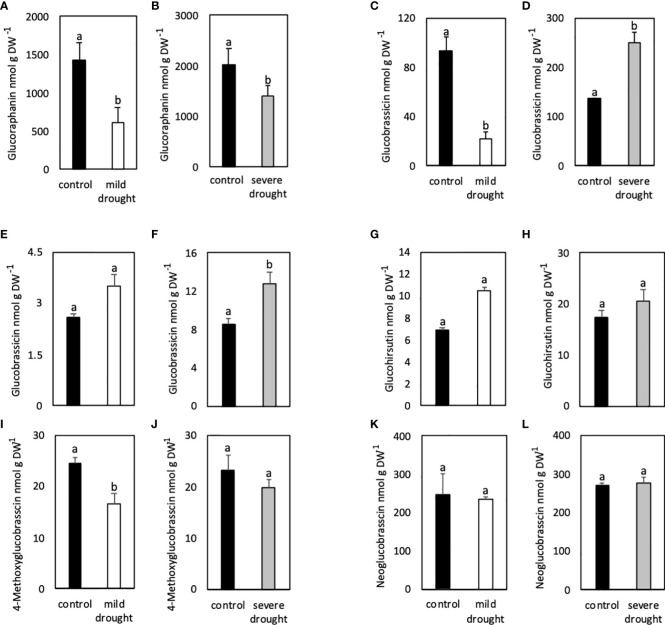
Glucosinolate contents in leaves **(A–D)** and roots **(E–L)** of mildly (white bars) and severely (grey bars) drought-stressed *Arabidopsis thaliana* Col-0 plants subjected to 20% and 40% PEG 20,000, respectively, for 7 days. Plants were grown on petri dishes for *five* weeks prior to application of drought stress. Bars represent means of three biological replicates consisting of four pooled plants each. Different letters indicate significant differences (p<0.05) between control and drought-stressed plants.

Contents of all GSLs in leaves of MDS plants were significantly lower compared to control plants (**Figures 2A, C**; **Supplementary Figure 4**). Contents of all aGSLs as well as the iGSLs 4-methoxyglucobrassicin and neoglucobrassicin were 2-3-fold lower in MDS compared to control plants (**Supplementary Figure 4**). On the other hand, contents of the iGSL GB were 4.5 -fold lower in MDS compared to control plants (**Figure 2C**).

Plants that were SDS, showed lower contents of all aGSLs (**Figure 2B**, **Supplementary Figure 5**) and lower contents of 4-methoxyglucobrassicin and neoglucobrassicin in leaves (**Supplementary Figures 5E, F**), whereas contents of GB were significantly higher in SDS compared to non-stressed control plants (**Figure 2D**).

Composition of GSLs in *A. thaliana* differed in roots. Glucoiberin, glucoalyssin, glucoerucin, and glucoraphanin were not detected in roots.

Glucobrassicin contents were 16-fold lower in roots (**Figure 2F**) compared to leaves (**Figure 2D**), whereas contents of neoglucobrassicin were two times higher in roots compared to leaves (**Figures 2K, L**; **Supplementary Figure 4F**; **Supplementary Figure 5F**). Glucobrassicin and glucohirsutin contents (**Figures 2E, G**) were slightly higher in roots of MDS compared to control plants, whereas contents of 4-methoxyglucobrassicin were significantly lower in MDS compared to control plants **Figure 2I**). Neoglucobrassicin contents were similar in control as well as both drought treatments (**Figures 2K, L**).

In roots, contents of GB were significantly higher in SDS compared to control plants (**Figure 2E**), whereas contents of glucohirsutin were just slightly higher in SDS plants (**Figure 2H**). Contents of 4-methoxyglucobrassicin were slightly lower in SDS compared to control plants (**Figure 2J**).

Analysis of GSLs showed that contents of GB behaved differently in MDS and SDS plants. While its contents were much lower in leaves of MDS plants, higher levels were observed in leaves of SDS plants. In SDS plants contents were significantly higher in all plant parts compared to controls.

### 3.3 Glucobrassicin showed the highest incorporation of deuterium

To be able to interpret GSL contents correctly and to ensure proper differentiation between breakdown and *de novo* biosynthesis, rate of GSL synthesis was investigated by analyzing incorporation of deuterium into GSL structures. Incorporation was achieved by subjecting control and DS plants to deuterium oxide (D_2_O). Total contents and GSL contents with incorporated deuterium (isotopomers) were compared to estimate GSL amounts synthesized during the time of deuterium exposure.

**Figure 3:**
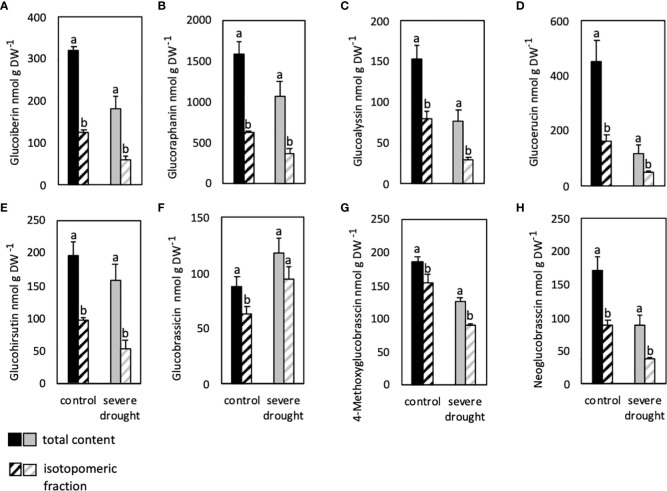
Isotopomeric and total contents of aliphatic **(A–E)** and indolic glucosinolates **(F–H)** in leaves of severely drought-stressed *Arabidopsis thaliana* Col-0 plants subjected to 40% PEG 20,000 and 30% D_2_O for seven days. Plants were grown on petri dishes for five weeks prior to application of severe drought stress (40% PEG 20,000). Bars represent means of three biological replicates consisting of four pooled plants each. Different letters indicate significant differences (p<0.05) between total glucosinolate contents and isotopomeric fractions.

The fraction of isotopomers compared to the total GSL content was significantly lower in all analyzed GSLs in leaves of control plants (**Figures 3A–H**). Nonetheless, fraction of isotopomers was much higher in the iGSLs GB and 4-methoxyglucobrassicin compared to isotopomeric fractions of aGSLs (**Figures 3F, G**). While the isotopmeric fraction of GB was 71% and 79% in control and SDS conditions respectively, the isotopomeric fraction of glucoraphanin was only 40 and 34% respectively. In SDS plants, fractions of isotopomers are significantly lower in all GSLs except for GB when compared to total contents (**Figure 3F**). Overall, total contents and isotopomeric fractions of GB were higher in SDS plants compared to controls much like SDS Col-0 plants that were not supplemented with D_2_O (**Figure 2D**, **Figure 3F**). Similarly, total contents of all other GSLs were lower in SDS plants compared to non-stressed controls in the same manner of plants not subjected to D_2_O (**Figure 2B**, **Supplementary Figure 5**, **Figures 3A–E, G, H**).

**Figure 4:**
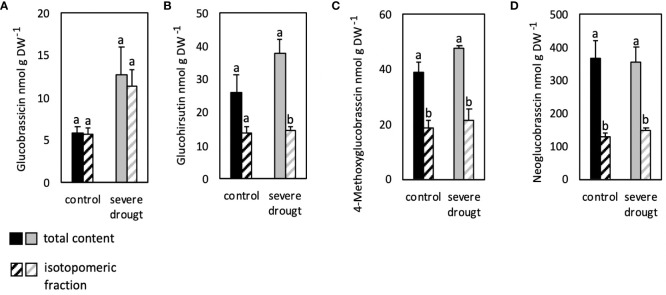
Isotopomeric and total contents of aliphatic **(B)** and indolic glucosinolates **(A, C, D)** in roots of severely drought-stressed *Arabidopsis thaliana* Col-0 plants subjected to 40% PEG 20,000 and 30% D_2_O for seven days. Plants were grown on petri dishes for five weeks prior to application of severe drought stress (40% PEG 20,000). Bars represent means of three biological replicates consisting of four pooled plants each. Different letters indicate significant differences (p<0.05) between total glucosinolate contents and isotopomeric fractions.

The fraction of isotopomers of glucohirsutin, 4-methoxyglucobrassicin and neoglucobrassicin analyzed in roots was significantly lower compared to the total GSL content in control and SDS conditions (**Figures 4B–D**). The isotopomeric fraction of GB on the other hand was similar to the total GSL content in both conditions (**Figure 4A**).

Overall, similar total and isotopomeric contents of GB showed highest deuterium incorporation into this particular GSL in all conditions and organs analyzed. Similarly, high incorporation of deuterium was observed in 4-methoxyglucobrassicin in leaves, but not roots.

### 3.4 Expression patterns correlated with stress intensity

Transcription analysis of control and DS plants was performed to gain insight into the expression of genes involved in transport and degradation of GSL and the modification of their breakdown products. Additionally, expression of *CYP71A13* was analyzed to estimate the contribution of the IAOX pathway to IAA synthesis in DS plants.

**Figure 5:**
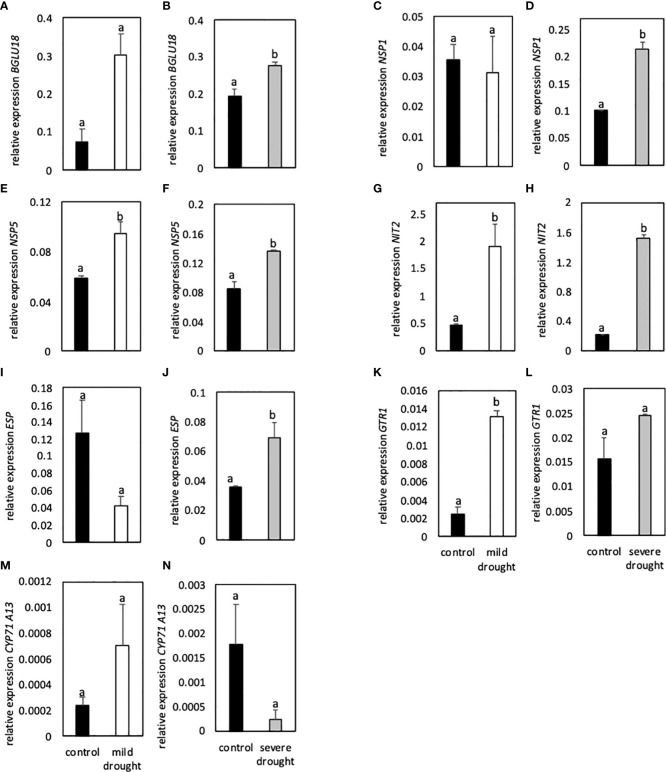
Relative expression of genes involved in glucosinolate breakdown (*BGLU18, NSP1, NSP5, ESP;* A-F, I-J), modification of breakdown products (*Nit2, G-H*), transport (*GTR1*; K-L) and synthesis of a key enzyme of indole-3-acetic acid synthesis (*CYP71A13*; M-N) to the reference gene *EF1α*. Plants were either subjected to 20% (mild drought stress, white bars) or 40% PEG 20,000 (severe drought stress, grey bars) for 7 days. Plants were grown on petri dishes for five weeks prior to application of drought stress. Bars represent means of three biological replicates consisting of four pooled plants each. Different letters indicate significant differences (p<0.05) between control and drought-stressed plants.

Expression of the thioglucosidase *BGLU18* was higher in MDS compared to control plants, but difference was only significant in SDS plants compared to controls (**Figures 5A, B**). Expression of *NSP1* was similar in MDS and control plants, whereas expression of *NSP5* was significantly higher in MDS plants (**Figures 5C, E**). In SDS plants, expression of *NSP1* and *NSP5* was significantly higher compared to control plants (**Figures 5D, F**). Expression of *NIT2* was significantly higher in both MDS and SDS when compared to control plants (**Figures 5G, H**). In SDS plants, expression of *ESP* was significantly higher compared to controls (**Figure 5J**).

Expression of *GTR1* was significantly higher in MDS and SDS plants compared to controls (**Figures 5K, L**). Expression of *CYP71A13* was significantly lower in SDS plants compared to controls (**Figure 5N**).

Expression of genes involved in breakdown (*BGLU18*), modification of breakdown products (*NSP1, NSP5, ESP*) and transport (*GTR1*) were significantly upregulated in DS plants compared to controls. Furthermore, expression of *CYP71A13* was significantly lower in SDS compared to control plants.

### 3.5 Selected mutants showed differences in contents of reactive oxygen species, glucobrassicin and expression of genes compared to Col-0

**Figure 6:**
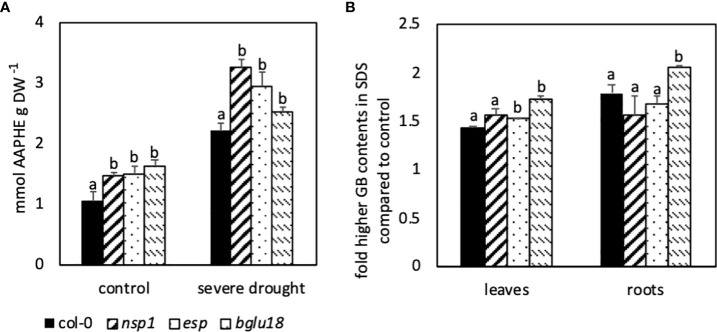
Differences in contents of reactive oxygen species (ROS) analyzed as 2,2′-azobis(2-amidino-propane) equivalents (AAPHE; **A**) and in fold higher contents of glucobrassicin (GB, **B**) in different mutants. Five-week-old *Arabidopsis thaliana* plants were subjected to severe drought stress (40% PEG 20,000) for 7 days. Bars represent means of two independent experiments consisting of three biological replicates consisting of four pooled plants each. Different letters indicate significant differences (p<0.05) between mutants and the wild-type Col-0.

To investigate the putative involvement of BGLU18 in the breakdown of iGSLs and the role of ESP in DS plants, mutants lacking these enzymes were analyzed in drought and control conditions. Analysis of ROS contents in *nsp1*, *esp* and *bglu18* revealed significantly higher contents in control and SDS plants when compared to Col-0 (**Figure 6A**). The differences in ROS content between mutants and wild-type were even more pronounced in SDS plants when compared to control plants.

The difference in GB contents in leaves between SDS and control plants analyzed in *esp* and *bglu18* was significantly larger when compared to Col-0, whereas *nsp1* showed only minor differences (**Figure 6B**). In roots on the other hand, the difference in GB contents between control and SDS plants was significantly larger in *bglu18* compared to all other genotypes.

**Figure 7:**
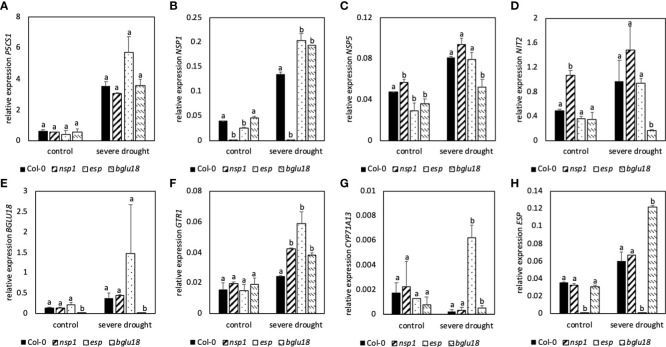
Relative expression of genes involved in stress response (P5CS1, A), glucosinolate breakdown (NSP1, B; NSP5, C; ESP, H; BGLU18, E), transport (GTR1, F) and synthesis of indole-3-acetic acid (NIT2, D;CYP71A13, G) to the reference gene EF1α. Plants were subjected to 40% PEG 20,000 (severe drought stress) for 7 days. Plants were grown on petri dishes for five weeks prior to application of drought stress. Bars represent means of three biological replicates consisting of four pooled plants each. Different letters indicate significant differences (p<0.05) between Col-0 and mutant plants.

Observation of *nsp1*, *esp* and *bglu18* revealed similar expression of *P5CS1* in control and SDS plants compared to Col-0 (**Figure 7A**). Expression of *NSP1* was higher in *esp* and *bglu18* in DS plants when compared to Col-0 (**Figure 7B**). Expression of *NSP1* in the *nsp1* mutant was barely detectable. On the other hand, expression of *NSP5* was significantly lower in *esp* and *bglu18* in control plants when compared to Col-0, while expression was only lower in SDS *bglu18* when compared to SDS control plants (**Figure 7C**). Expression of *NIT2* was significantly lower in SDS *bglu18* in SDS plants when compared to Col-0, while only *nsp1* showed higher contents in control conditions when compared to Col-0 (**Figure 7D**). Expression of *GTR1* was similar in mutant control plants when compared to Col-0, whereas expression was significantly higher in SDS *nsp1*, *esp* and *bglu18* when compared to SDS Col-0 (**Figure 7F**). In SDS plants, expression of *CYP71A13* was significantly higher in *esp* and *bglu18* compared to Col-0 (**Figure 7G**). Expression of *ESP* was significantly lower in *esp* in all conditions, whereas expression in *bglu18* was significantly higher in SDS plants when compared to Col-0 (**Figure 7H**).

Compared to Col-0, *nsp1*, *esp* and *bglu18* revealed higher contents of ROS and *esp* and *bglu18* showed higher induction of GB contents in DS conditions compared to control plants. Furthermore, expression of *NSP1*, *GTR1* and *CYP71A13* were higher in *esp* and *bglu18* compared to Col-0. Interestingly, expression of *BGLU18* was higher in *esp* and expression of *ESP* was higher in *bglu18* when compared to Col-0 (**Figure 7E**).

**Figure 8:**
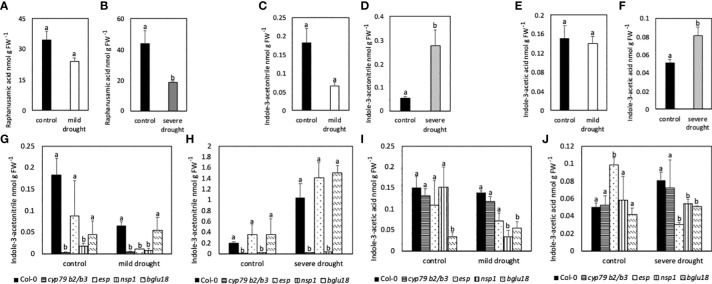
Contents of raphanusamic acid (RA; **A, B**), indole-3-acetonitrile (IAN; **C, D, G, H**) and indole-3-acetic acid (IAA, **E, F, I, J**) in leaves of mildly and severely drought-stressed *Arabidopsis thaliana* Col-0 plants and *cyp79B2/B3* and *nsp1* mutants subjected to 20% and 40% PEG 20,000 for seven days. Plants were grown on petri dishes for five weeks prior to application of drought stress. Bars represent means of three biological replicates consisting of four pooled plants each. Different letters indicate significant differences (p<0.05) between control and drought-stressed plants **(A–F)** and between Col-0 and mutants **(G–J)**.

Contents of the GB breakdown product RA were similar in MDS and control plants, whereas contents were significantly lower in SDS compared to control plants (**Figures 8A, B**). Contents of IAN were lower in MDS when compared to control plants, but significantly higher in SDS compared to control plants (**Figures 8C, D**). Compared to Col-0, *cyp79B2/B3* and *nsp1* mutants had significantly lower contents of IAN in all conditions, while *esp* showed lower contents only in drought stressed conditions. Contents of IAN were similar in *bglu18* when compared to Col-0 (**Figures 8G, H**). In all samples the amount of IAA was below the reliable detection limit. Nevertheless, contents of IAA are shown in **Figures 8E, F, I, J**. Contents of IAA were similar in MDS (**Figure 8E**), but significantly higher in SDS compared to control plants (**Figure 8F**). Contents were significantly lower in MDS and SDS *esp, bglu18* and *nsp1* mutants compared to Col-0 (**Figures 8I, J**).

## 4 Discussion

### 4.1 Results obtained from plants grown on soil can be replicated *in vitro*

After establishment of a reliable drought stress treatment for *A. thaliana* grown on soil (**Supplementary Figure 7**, **Supplementary Figure 8**), an *in vitro* cultivation method was developed to facilitate the harvest of roots. Mild and severe drought stress were applied by subjecting five-week-old plants to 20% and 40% PEG 20,000, respectively, for 7 days.

To draw conclusions about the desiccation status of plants, the leaf water content was analyzed, clearly showing lower leaf water contents in SDS compared to MDS plants (**Supplementary Figure 2**). Nonetheless, leaf water content was significantly lower in both conditions when compared to controls, a clear indication of water loss in both DS conditions.

In order to analyze the amount of drought-induced ROS emerging in the plant, a fast and reliable photometric assay was developed. The higher amount of ROS in DS compared to control plants shows the successful induction of stress in the plants. Elevated levels of ROS were previously reported in DS plants and could therefore be used as reliable DS marker (Qi et al., 2018).

P5CS1 is the rate limiting key enzyme in proline synthesis and therefore a marker for drought stress (Chen et al., 2018). Expression of *P5CS1* is highly elevated in SDS plants *in vitro* (**Figures 1H, J**, in plants grown on soil (**Supplementary Figure 8**) and MDS plants (**Supplementary Figure 1E**) when compared to control plants. Higher expression of *P5CS1* in drought conditions was previously described in the literature for *A. thaliana* (Zhang et al., 2016). *In vitro*, expression of *P5CS1* positively correlates with the strength of drought application. Similarly, observations in *Hordeum vulgare* done by Muzammil et al. (2018) showed a positive correlation between duration of drought stress and expression of *P5CS1* indicating higher expression in plants exposed to more severe drought stress. In plants grown on soil on the other hand, expression of *P5CS1* in DS plants is much higher compared to DS plants grown *in vitro*. This might be explained by gradual desiccation of plants on soil compared to instant application of PEG *in vitro*. Soil-grown and drought-stressed plant are therefore exposed to a longer duration of drought stress. Furthermore, plants grown on soil are subjected to higher water loss from stomata compared to *in vitro* plants growing in a humid microclimate.

Nonetheless, most changes in GSL contents and changes in transcription levels observed in plants grown on soil were successfully replicated *in vitro* supporting the reliable establishment of drought stress conditions on soil and *in vitro*.

Lack of chlorosis and senescence indicated that application of drought stress did not lead to irreparable damage to the plants. Furthermore, experiments performed on soil (**Supplementary Figures 7**, **8**) showed more expression of *P5CS1* suggesting a stronger application of drought stress without being detrimental to the plants’ overall health.

### 4.2 Glucosinolates get broken down during drought stress in *Arabidopsis thaliana*

Contents of all GSLs in leaves were significantly lower in MDS compared to control plants (**Figure 2**). Similarly, contents of all GSLs except GB were lower in SDS plants compared to control plants (**Figure 2**). Lower GSL levels indicate either lower synthesis rates or breakdown that exceeds the *de novo* biosynthesis.

Incorporation of deuterium into GSLs of SDS plants revealed that only a fraction of the total GSL content of aGSL and neoglucobrassicin was found to have deuterium incorporated. This shows that most of the total content was synthesized before the administration of D_2_O (**Figure 3**). Isotopomeric fractions of aGSLs ranged from 32% - 42% and were 44% for neoglucobrassicin clearly revealing lower biosynthesis rates in SDS compared to control plants.

On the other hand, isotopomeric contents of GB were higher in SDS (80%) compared to control plants (71%). The high incorporation of deuterium into GB shows the increase of an already high synthesis and therefore underlines the need for this particular compound, especially in SDS plants. Furthermore, low contents of GB without incorporation show a high breakdown in control and DS conditions. Both factors highlight the high turnover of GB and indicate the need for a constant supply of GB derived breakdown products. Furthermore, 4.5-times lower contents of GB in MDS compared to control plants points to increased degradation of GB in MDS plants. The turnover of GB probably exceeds the *de novo* biosynthesis in MDS compared to the higher synthesis in SDS plants.

Although expression of classical thioglucosidases *TGG1* and *TGG2* was higher in SDS compared to control plants (**Supplementary Figure 15**), their contribution to GSL turnover in intact tissue is still a matter of debate (Meier et al., 2019). While TGG1 and TGG2 are transported to vacuoles of myrosin cells (Ueda et al., 2006), the final step of GSL biosynthesis takes place in the cytosol (Klein et al., 2006). Instead, the atypical thioglucosidases PYK10 and PEN2 were identified to be responsible for the turnover of GSLs in undisrupted tissues. PYK10 is localized in ER-bodies (Nakano et al., 2017), and PEN2, is localized in peroxisomes (Bednarek et al., 2009), potentially placing them into close proximity to GSLs. BGLU18, was also found to be localized in ER-bodies (Nakazaki et al., 2019), but its contribution to GSL turnover is still a hypothesis.

### 4.3 The proteins BGLU18 and ESP, NSP and the metabolite glucobrassicin are tightly interconnected

**Figure 9:**
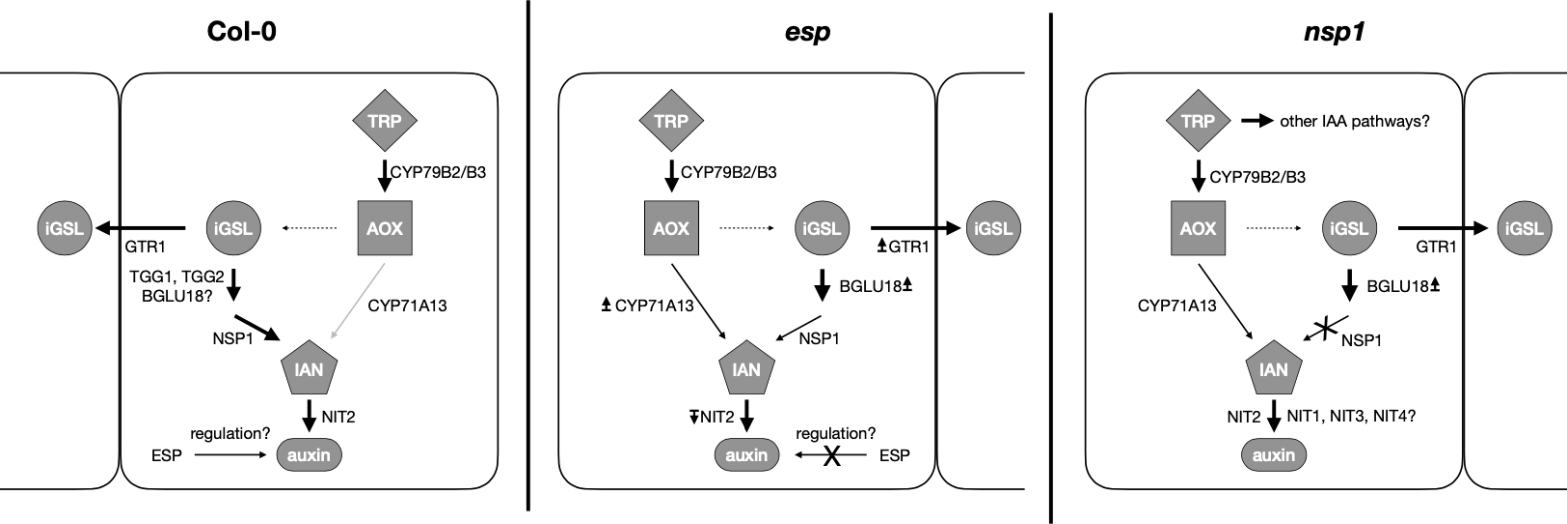
Expression patterns in control and drought-stressed Col-0, *esp* and nsp1 *Arabidopsis thaliana* plants. TRP=tryptophan, AOX=Indole-acetaldoxime, iGSL=indolic glucosinolates, IAN=indole-3-acetonitrile, auxin=iGSL derived carboxylic acids. Bold arrows indicate significantly higher expression, light grey arrows indicate lower expression in severely drought-stressed compared to control plants. Arrows near enzymes indicate higher (↥) or lower (↧) expression of genes compared to Col-0. Dashed arrow indicates synthesis steps involving multiple genes. In Col-0 genes expressing proteins responsible for the degradation of glucobrassicin and converting its breakdown products are highly expressed in drought-stressed plants compared to controls. In *esp*, auxin synthesis is redirected towards synthesis directly from AOX. Unchanged expression of *CYP71A13* and *NIT2* in *nsp1* mutants indicates the involvement of other nitrilases leading to depletion of IAN or complete redirection of IAA synthesis to other pathways.

Significantly higher expression of *BGLU18* in MDS and SDS compared to control plants suggests the putative involvement of BGLU18 in the breakdown of GSLs (**Figures 5A, B**). BGLU18 is primarily known for the production of abscisic acid from the abscisic acid glycosyl ester and higher expression of *BGLU18* is shown in stress situations such as drought (Sugiyama & Hirai, 2019; Han et al., 2020). The *bglu18pyk10* double mutant showed reduced breakdown of 4-methoxyglucobrassicin in homogenized plant material of *A. thaliana* indicating the involvement of either PYK10 or BGLU18 (Nakazaki et al., 2019). Although levels of GB were unaltered upon homogenization of tissue in the study of Nakazaki et al. (2019), the involvement of BGLU18 in the breakdown of all iGSL in intact tissue cannot be excluded. Contents of GB (**Figures 2C, D**), its turnover (**Figure 3F**) and simultaneous expression of *BGLU18, NSP1 and NSP5* were significantly higher in leaves of SDS compared to control plants. This suggests the putative breakdown of GB by BGLU18 and subsequent formation of nitriles by specifier proteins. Similar contents of IAN in bglu18 mutants when compared to Col-0 (**Figures 8G, H**) indicate the compensation of iGSL breakdown by other thioglucosidases (e.g. TGG1, TGG2). Lower contents of IAA in DS *bglu18* compared to DS Col-0 similarly to contents observed in DS *esp* (**Figures 9I, J**), indicates a codependence of both enzymes which is reflected in the transcription levels of both enzymes in the mutants (**Figures 7E, H**). However, the certain involvement of BGLU18 in the breakdown of glucosinolates could not be demonstrated and the involvement of other thioglucosidases should be taken into consideration. Because ESP protein was not yet detected in *A. thaliana* Col-0, its involvement in the formation of IAN from GB is unlikely (Kissen et al., 2012). However, presence of small undetectable quantities of ESP cannot be excluded. Miao and Zentgraf (2007) reported regulatory activity of ESP which would require only minute amounts of protein. The study observed reduced leaf senescence upon interaction of ESP with the transcription factor WRKY63 in Col-0. The interaction requires the presence of an ESP protein and therefore strongly suggests the presence of ESP in Col-0. A connection between ESP, WRKY63 and GSLs was not yet established but possible targets of the transcription factor could be genes involved in the synthesis and breakdown of GB and in the synthesis of IAA. Comparing the differences in GB contents between control and SDS plants in fold changes revealed significantly higher values in leaves of *esp* and in leaves and roots of *bglu18* mutants when compared to Col-0. Furthermore, SDS Col-0 showed higher contents of IAA (**Figure 8J**). However, *esp* mutants seem to be unable to synthesize IAA to the same extent as Col-0. This further indicates, that SDS *esp* mutants are compromised in their ability to synthesize IAA when compared to Col-0. However, it is unclear at this moment why *esp* mutants exhibit altered IAA contents. Unaltered IAN contents in *esp* mutants compared to Col-0 indicates that the effect of missing ESP is not due to a direct enzymatic but rather an indirect regulatory function.

Additionally, *esp* and *bglu18* mutants showed significantly higher contents of ROS in control and SDS conditions when compared to Col-0 (**Figure 6A**), suggesting a higher stress status due to lack of either enzyme. Better management of ROS accumulation in drought-stressed plants was shown to improve stress tolerance of crops (You and Chan, 2015; Park et al., 2019; Nadarajah, 2020). Therefore, BGLU18, ESP and NSP1 could be potential targets in the selection of more drought tolerant Brassica crops.

### 4.4 Indole-3-acetonitrile is formed during severe drought

In a study done by Wittstock & Burow (2010), NSPs were shown to aid in nitrile formation upon the breakdown of iGSLs. In another research done by Wittstock et al. (2016), it was shown that *NSP1* and *NSP5* were expressed in leaves, whereas *NSP3-NSP4* were only expressed marginally indicating a higher contribution of NSP1 and NSP5 to nitrile formation in leaves. Significantly higher expression of *NSP5* in MDS compared to control plants indicates increased formation of nitriles from GSLs already in mild drought conditions (**Figure 5**). However, similar contents of IAN in MDS and control plants and higher expression of *NIT2* indicate the further conversion of IAN to IAA in MDS plants (**Figure 7D**). In addition to *NSP5*, *NSP1* is significantly higher expressed in SDS compared to control plants, suggesting an increased need of nitrile formation in SDS compared to MDS plants. Significantly higher contents of IAN in SDS and significantly lower contents of RA compared to control plants indicate the favored synthesis of nitriles rather than ITCs in SDS plants (**Figures 8B, D**). In line with published data of Zhao et al. (2002) and Sugawara et al. (2009), barely detectable contents of IAN in *cyp79B2/B3* mutants shows that IAN is mainly synthesized *via* the CYP79B2/B3 pathway. Contents of IAN (**Figures 8E, F**) were very low in *nsp1* mutants indicating a major contribution of NSP1 to IAN formation in line with published data (Wittstock et al., 2016; Dörr, 2017). Furthermore, lower expression of *CYP71A13* (**Figure 7G**) and unaltered expression of *NIT2* (**Figure 7D**) in SDS *nsp1* mutants compared to control plants indicates the redirection of IAA synthesis to pathways independent of IAOX. In line with this, research of Sugawara et al. (2009) showed that IAN contents were unaltered in cyp71A13 mutants growing under standard conditions indicating the bypass of IAA synthesis by other pathways. Furthermore, IAA synthesis pathways with indole-3-pyruvic acid were hypothesized to be the main IAA synthesis pathway, at least under standard growing conditions (Mashiguchi et al., 2011). Since *cyp79B2/B3* mutants did not exhibit phenotypical alterations in any tested conditions (**Supplementary Figure 17**) loss of the ability to synthesize IAA thought the CYP79B2/B3 pathway is not reflected in major growth alterations.

However, expression of *CYP71A13* was significantly higher in both SDS *esp* and *bglu18* mutants compared to Col-0 (**Figure 7G**). This clearly indicates the redirection of auxin biosynthesis towards the aldoxime pathway mediated by CYP71A13 (**Figure 9**) bypassing the compromised iGSLs pathway.

Overall, *nsp* mutants probably redirect IAA synthesis through pathways completely independently of IAOX. However, *esp* and *bglu18* mutants compensate for the compromised GB breakdown machinery by synthesizing IAN through *CYP71A13* directly.

### 4.5 Glucobrassicin-derived breakdown products are probably converted to indole-3-acetic acid

Four nitrilase genes *NIT1*-*NIT4* are encoded in the genome of *A. thaliana*. While NIT4 was shown to detoxify hydrogen cyanide, the *NIT1*-subfamily (*NIT1*-*NIT3*) seems to have more far reaching functions like protection against pathogens, involvement in senescence and root morphology during sulfur deprivation (Lehmann et al., 2017). Additionally, NIT1-3 were shown to convert IAN to IAA (Vorwerk et al., 2001) connecting the breakdown of iGSLs to the biosynthesis of auxin (Malka & Cheng, 2017). Significantly higher expression of *NIT2* in MDS and SDS compared to control plants clearly shows its importance in DS plants and its putative involvement in the synthesis of carboxylic acids from iGSLs (**Figure 5G, H**). From the NIT1-subfamily NIT2 was shown to have the highest affinity towards indole-3-acetonitrile, hinting to NIT2 being more involved in the formation of carboxylic acids from iGSLs than other nitrilases (Vorwerk et al., 2001). Higher contents of GB, IAN and IAA (**Figures 8D, F**) in SDS plants compared to controls suggests the synthesis of IAA from the GB pathway. Additionally, higher expression of *NSP5* and *NIT2* further corroborate this assumption. Higher contents of IAA in DS plants as illustrated in **Figure 8F** could lead to the increased formation of lateral roots and the subsequent acquisition of water in the root zone. Lateral root formation and enhanced drought tolerance after application of exogenously applied IAA was observed by Shi et al. (2014). Lower contents of IAA in DS *esp* and *nsp1* mutants as compared to Col-0 (**Figure 8J**) could therefore lead to lower drought tolerance and subsequently higher contents of ROS as shown in **Figure 6A**).

### 4.6 Glucosinolate contents differ in roots and shoots

Higher incorporation of deuterium into GB compared to other GSLs clearly shows the importance of this compound in leaves (**Figure 3A**) and roots (**Figure 4A**) as almost the complete content was synthesized since the administration of deuterium. Nevertheless, contents of GB are much lower in roots compared to leaves (**Figure 2**). Since GB is the parent GSL to 4-methoxyglucobrassicin and neoglucobrassicin, a conversion seems evident (Pfalz et al., 2011). CYP81F4 is responsible for the conversion of GB to neoglucobrassicin. Expression of *CYP81F4* is much higher in roots (Pfalz et al., 2016), but contents of neoglucobrassicin are also found in leaves raising the question about the contribution of GSL transport.

The GSL transporters GTR1 and GTR2 are known to relocate GSLs into different cells and organs (Jørgensen et al., 2015; Chhajed et al., 2019). They are known to be highly expressed during bolting and seed filling, being responsible for relocation of GSLs to seeds. Plants were neither bolting nor flowering (**Supplementary Figures 2G–J**), but expression of *GTR1* was significantly higher in MDS and SDS compared to control plants. Therefore, GSLs relocation seems to be important in drought-stressed plants. Significantly higher expression of *GTR1* in SDS *bglu18* and *esp* mutants compared to SDS Col-0 (**Figure 7F**) further underlines the enhanced need for iGSL relocation. However, the need for relocation of iGSLs raises the question why different iGSLs are needed in separate organs of the plants.

## 5 Conclusion

In this study, we demonstrate that although *A. thaliana* has a multitude of pathways to yield IAA from, several genes from the iGSL pathway yielding IAA are highly expressed in SDS plants compared to controls. Through deuterium incorporation studies it was shown, that during SDS, GB seems to be one of the most important GSLs, since its contents showed the highest turnover of all analyzed GSLs. Furthermore, the higher level of expression of genes involved in synthesis (*cyp79B2/B3*), breakdown (*BGLU18*, *NSP1*, *NSP5*, *ESP*) and relocation (*GTR1*) of iGSLs and synthesis of IAN (*NIT2*) from which IAA is most likely being formed, strongly suggest the importance of this particular pathway in drought stress compared to control conditions. Finally, we can show that a lack of either BGLU18 or ESP seems to be redirecting auxin biosynthesis to other pathways, including synthesis of IAA directly from IAOX.

## Data availability statement

The original contributions presented in the study are included in the article/**Supplementary Materials**. Further inquiries can be directed to the corresponding author.

## Author contributions

JH, IH-N and JP conceived and designed the experiments. JH performed the experiments. Glucosinolate analysis and assessment of stress status was performed by JH. Analysis of raphanusamic acid, indole-3-acetonitrile and indole-3-acetic acid was done by CH and IF. Transcription analysis was performed by IH-N. Sorting of data, graphical design and statistical analysis was performed by JH. Manuscript was written by JH. IH-N, CH, IF and JP discussed and commented on results and manuscript. JP supervised the study. All authors contributed to the article and approved the submitted version.

## Funding

IF acknowledges funding through the German Research Foundation (DFG, INST 186/822-1).

## Acknowledgments

We are grateful for Sabine Freitag for technical assistance.

## Conflict of interest

The authors declare that the research was conducted in the absence of any commercial or financial relationships that could be construed as a potential conflict of interest.

## Publisher’s note

All claims expressed in this article are solely those of the authors and do not necessarily represent those of their affiliated organizations, or those of the publisher, the editors and the reviewers. Any product that may be evaluated in this article, or claim that may be made by its manufacturer, is not guaranteed or endorsed by the publisher.

## Abbreviations

DS, Drought-stressed; ESP, Epithionitrile specifier protein; GB, Glucobrassicin; GSL, Glucosinolate; IAA, Indole-3-acetic acid; IAN, Indole-3-acetonitrile; IAOX, Indole-3-acetaldoxime; ITC, Isothiocyanate; MDS, Mildly drought-stressed; NSP, Nitrile specifier protein; SDS, Severely drought-stressed.
