# Supplementary material for: First experimental evidence suggests use of glucobrassicin as source of auxin in drought-stressed Arabidopsis thaliana
Source: Front Plant Sci. 2022 Oct 31;13:1025969. doi: 10.3389/fpls.2022.1025969 (PMC9659865; doi:10.3389/fpls.2022.1025969)
Supplement: Supplementary file 1 [file DataSheet_1.pdf]

Supplementary data

Supplementary Table S1 Primer sequences for qPCR of all analyzed genes mentioned in this study and qPCR validation data.

TAIR code	Gene name	Forward 5‘->3‘	Reverse 5‘->3‘	slope	y-intercept	efficiency	R^2	Amplicon length
At5g60390	*EF1a*	CCAAGGCTAGGTACGATGAAATC	CGAATCCAGAGATTGGCACAAATG	-3.141	35.444	108%	0,997	102 bp
At4g05320	*UBQ10*	GGCCTTGTATAATCCCTGATGAATAAG	AAAGAGATAACAGGAACGGAAACATAGT	-3.29	37.668	101%	0,999	61 bp
At2g39800	*P5CS1*	GGAAAAGGACAAGTTGTCGACG	CAAATACACAAACACACACTCGGAAG	-3.307	37.029	100%	0.999	104 bp
At3g16400	*NSP1*	AGACCTGGGGTTAAGTTTGTGCT	CCCCAAGGGAGTGTAAAACATCTG	-3.299	36.14	100%	0.999	85 bp
At5g48180	*NSP5*	GGGAGAGGATTGTGTGCGGG	CAAGCCGCTCATTGGTCGGA	-3.422	34.26	96%	0.999	135 bp
At3g44300	*NIT2*	GCACCCGAAGAAACCGGTCA	TGAACTGACGAATCACAACCGA	-3.379	36.006	97%	0.997	89 bp
At1g52400	*BGLU18*	GAACGTTACGGGATACTTTGTGTG	GAATTCGGAATACCATTTGCCCG	-3.409	39.887	97%	0.997	118 bp
At3g47960	*GTR1*	CGTCGCTCAGAAGAGTGACCG	ATTGATGAGATTTCTCCTGTCCGAG	-3.513	35.805	93%	0.997	184 bp
At2g30770	*CYP71A13*	AAGTGAGTCGAGGGTTTAGCG	CGAAATCCGCTTTATCGTTACTCG	-3.205	35.826	105%	0.995	81 bp
At1g54040	*ESP*	TGCGTTGGACACCGAGACAC	GCCATGCATGAGGAGGCCAT	-3.401	35.537	97%	0.996	127 bp
At5g26000	*TGG1*	CCAGGTGATGAGGACTTTGAG	CACTTGATGACTTTACTGAGGAAAC	-3.234	37.116	103%	1	95 bp
At5g25980	*TGG2*	GTGGCCCCATACCCTTCAC	CACTTGATGGCCTTGCGGAG	-3.393	36.248	97%	1	94 bp

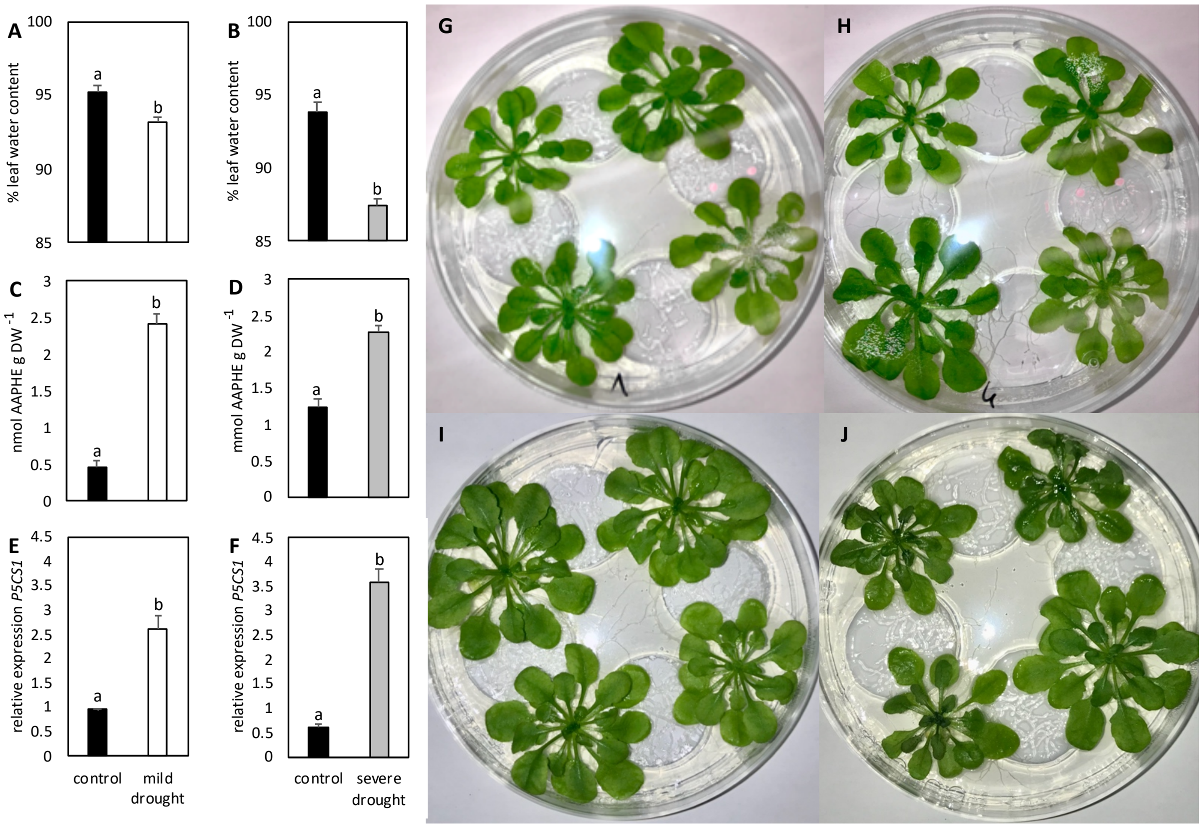

Supplementary Figure S2 Indicators of stress analyzed in *Arabidopsis thaliana* Col-0 subjected to mild or severe drought stress for 7 days. Plants were grown on petri dishes for five weeks prior to application of drought stress. A-B: Total leaf water content; C-D: Content of reactive oxygen species (ROS) analyzed as 2,2′-azobis(2-amidino-propane) equivalents (AAPHE); E-F: Expression of the drought-induced gene *P5CS1* relative to the standard gene *EF1α*; G,I: Control plants, H: Mildly drought-stressed plants subjected to 20% PEG 20,000 for 7 days; J: Severely drought-stressed plants subjected to 40% PEG 20,000 for 7 days. Bars represent means of three biological replicates consisting of four pooled plants each. Different letters indicate significant differences (p<0.05) between control and drought-stressed plants.

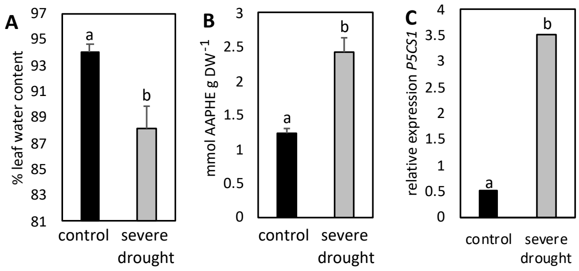

Supplementary Figure S3 Repetition of severe drought-stress experiment (Supplementary Fig. 2). Indicators of stress analyzed in *Arabidopsis thaliana* Col-0 subjected severe drought stress for seven days. Plants were grown on petri dishes for five weeks prior to application of severe drought stress (40% PEG 20,000). A: Total leaf water content; B: Content of reactive oxygen species (ROS) analyzed as 2,2′-azobis(2-amidino-propane) equivalents (AAPHE); C: Expression of the drought-induced gene *P5CS1* relative to the standard gene *EF1α*. Bars represent means of three biological replicates consisting of four pooled plants each. Different letters indicate significant differences (p<0.05) between control and drought-stressed plants.

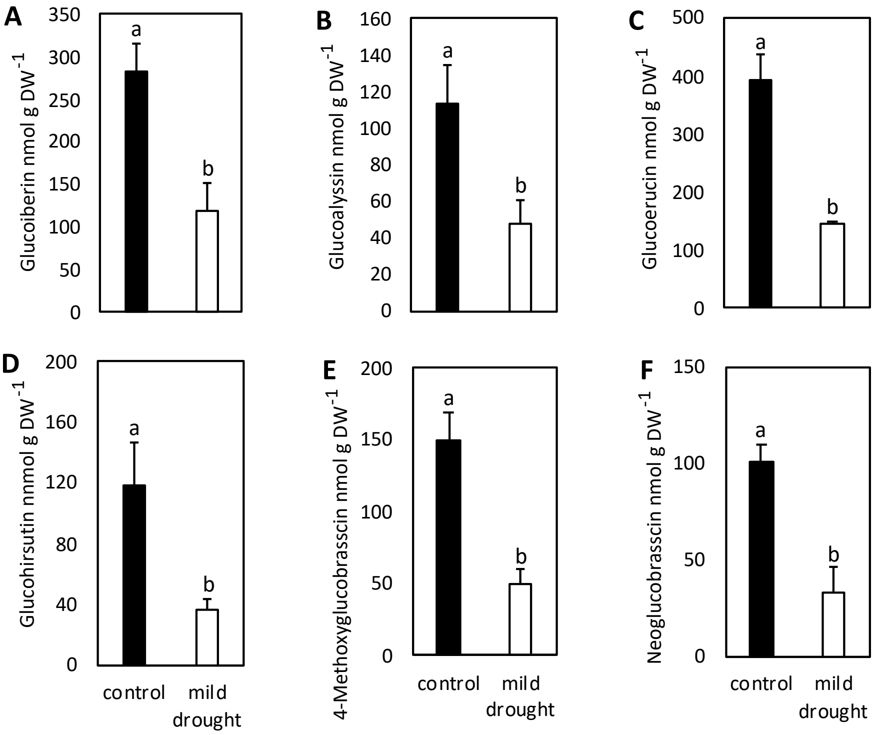

Supplementary Figure S4 Remaining contents of aliphatic (A-D) and indolic glucosinolates (E-F) in leaves of mildly drought-stressed *Arabidopsis thaliana* Col-0 plants subjected to 20% PEG 20,000 for seven days (Fig. 2). Plants were grown on petri dishes for five weeks prior to application of mild drought stress (20% PEG 20,000). Bars represent means of three biological replicates consisting of four pooled plants each. Different letters indicate significant differences ($p<0.05$) between control and drought-stressed plants.

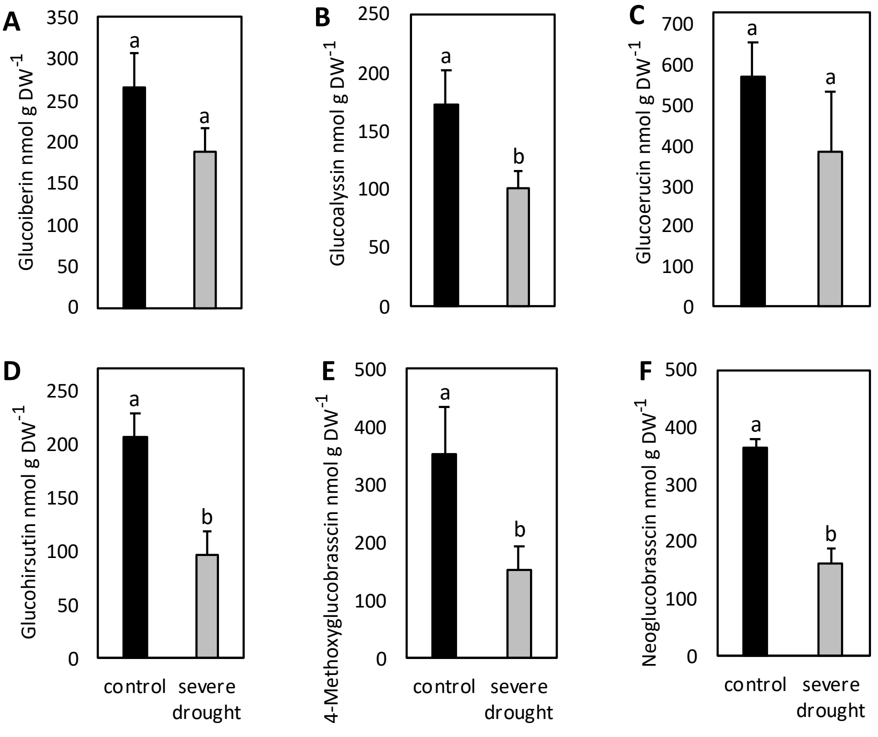

Supplementary Figure S5 Remaining contents of aliphatic (A-D) and indolic glucosinolates (E-F) in leaves of severely drought-stressed *Arabidopsis thaliana* Col-0 plants subjected to 40% PEG 20,000 for seven days (Fig. 4). Plants were grown on petri dishes for five weeks prior to application of severe drought stress (40% PEG 20,000). Bars represent means of three biological replicates consisting of four pooled plants each. Different letters indicate significant differences ($p<0.05$) between control and drought-stressed plants.

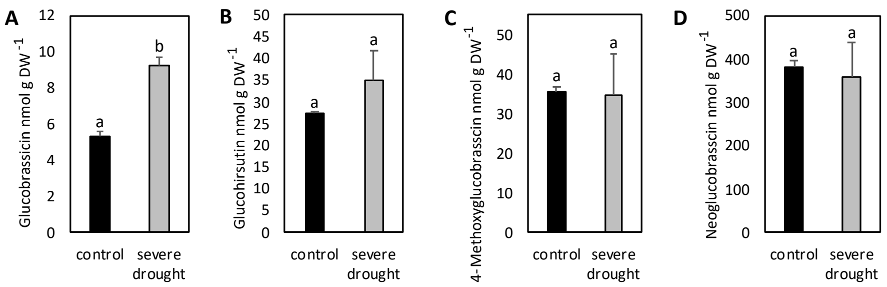

Supplementary Figure S6 Repetition of severe drought-stress experiment (Fig. 4). Aliphatic (B) and indolic (A, C, D) glucosinolate contents in roots of severely drought-stressed *Arabidopsis thaliana* Col-0 plants subjected to severe drought stress (40% PEG 20,000) for seven days. Plants were grown on petri dishes for five weeks prior to application of drought stress. Bars represent means of three biological replicates consisting of four pooled plants each. Different letters indicate significant differences ($p<0.05$) between control and drought-stressed plants.

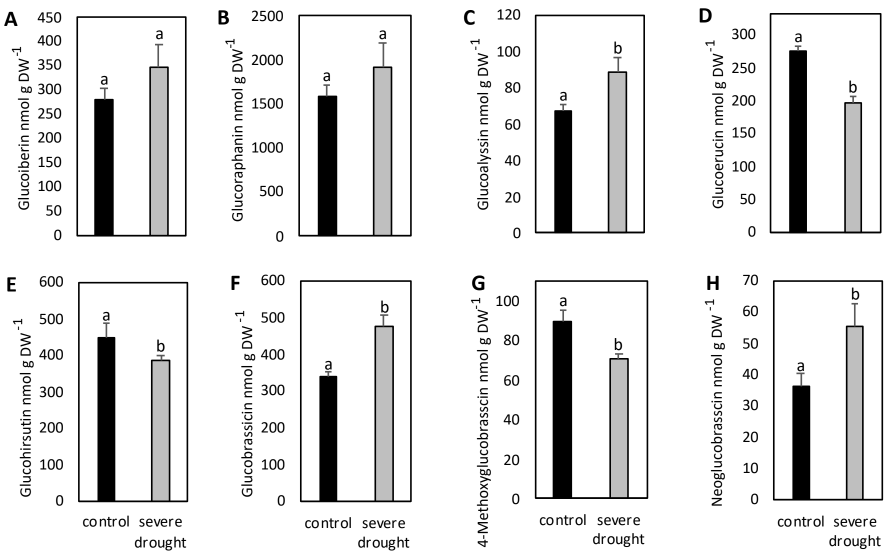

Supplementary Figure S7 Preceding severe drought-stress experiment with plants grown on soil. Aliphatic (A-E) and indolic (F-H) glucosinolate contents in leaves of severely drought-stressed *Arabidopsis thaliana* Col-0 plants subjected to 40% soil water content for five days. Plants were grown with optimal water supply for five weeks prior to application of drought stress. Bars represent means of three biological replicates consisting of three pooled plants each. Different letters indicate significant differences ($p<0.05$) between control and drought-stressed plants.

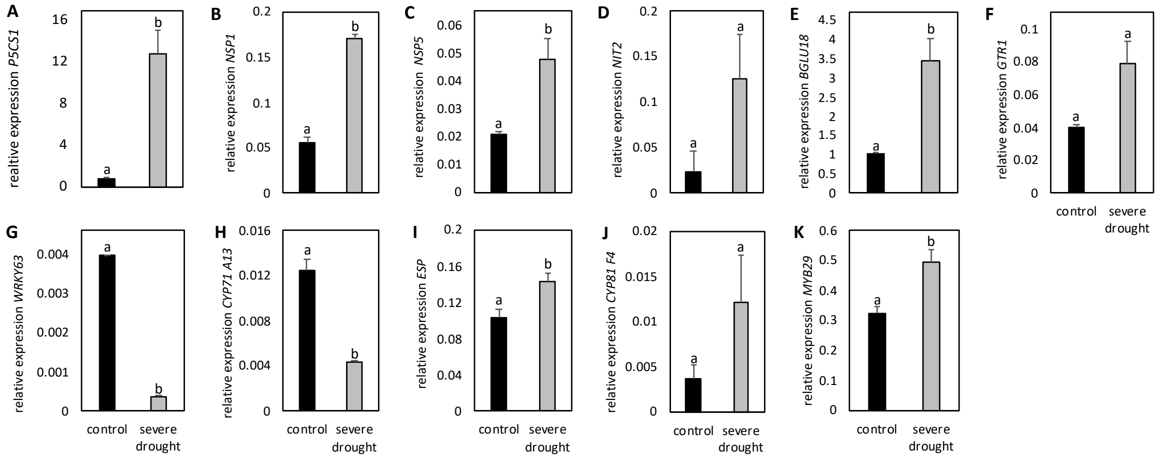

Supplementary Figure S8 Preceding severe drought-stress experiment with plants grown son soil. Relative expression of genes involved in water homeostasis (*P5CS1, A*), glucosinolates breakdown (*NSP1, B; NSP5, C; BGLU18, E*), transport (*GTR1, F*), regulation (*WRKY63, G; MYB29, K*), synthesis (*CYP81F4, J*) and synthesis of indole-3-acetic acid (*NIT2, D; CYP71A13, H*) to the reference gene EF1α. *Arabidopsis thaliana* Col-0 plants were subjected to 40% soil water content for five days. Plants were grown with optimal water supply for five weeks prior to application of drought stress. Bars represent means of three biological replicates consisting of three pooled plants each. Different letters indicate significant differences ($p<0.05$) between control and drought-stressed plants.

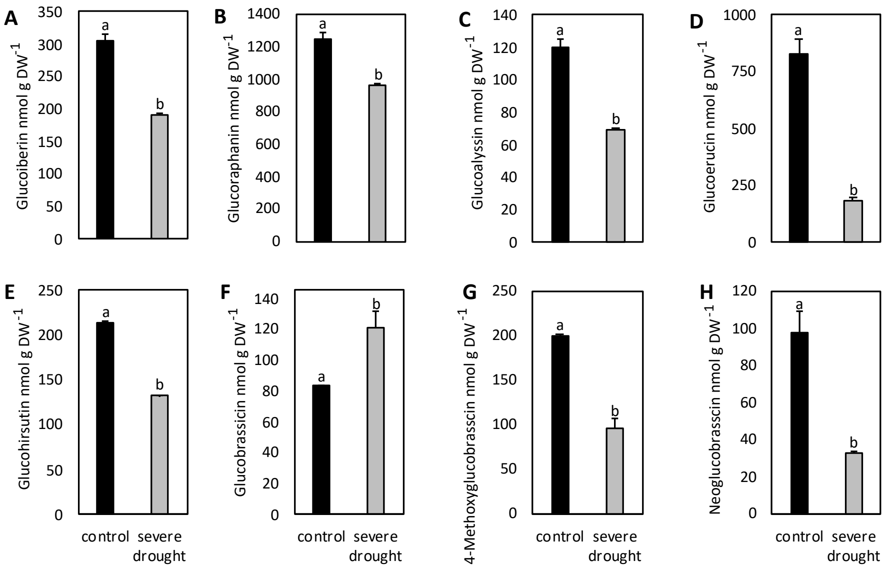

Supplementary Figure S9 Repetition of severe drought-stress experiment (Fig. 3). Aliphatic (A-E) and indolic (F-H) glucosinolate contents in leaves of severely drought-stressed *Arabidopsis thaliana* Col-0 plants subjected to severe drought stress (40% PEG 20,000) for seven days. Plants were grown on petri dishes for five weeks prior to application of drought stress. Bars represent means of three biological replicates consisting of four pooled plants each. Different letters indicate significant differences ($p<0.05$) between control and drought-stressed plants.

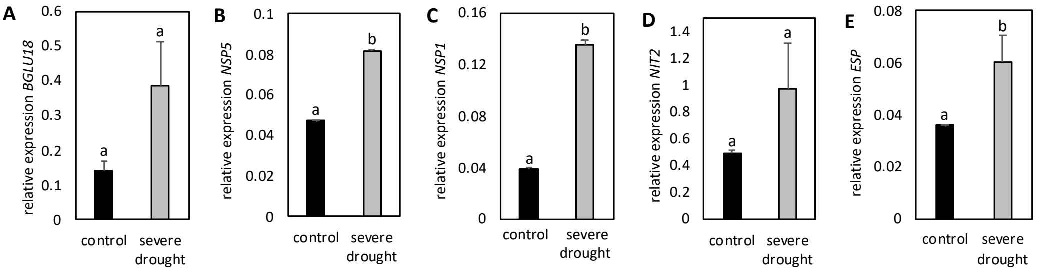

Supplementary Figure S10 Repetition of severe drought-stress experiment (Fig. 7). Relative expression of genes involved in glucosinolates breakdown (*BGLU18, A; NSP5, B; NSP1, C; ESP, E*) and modification (*Nit2, D*) of breakdown products to the reference gene *EF1α*. Plants were subjected 40% PEG 20,000 (severe drought stress) for seven days. Plants were grown on petri dishes for five weeks prior to application of drought stress. Bars represent means of three biological replicates consisting of four pooled plants each. Different letters indicate significant differences (p<0.05) between control and drought-stressed plants.

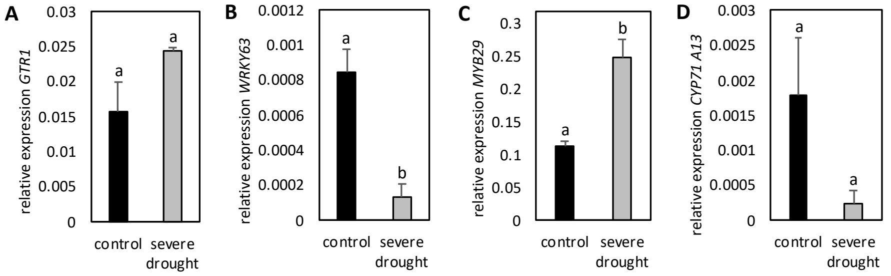

Supplementary Figure S11 Repetition of severe drought-stress experiment (Fig 8). Relative expression of genes involved in transport (*GTR1, A*) and regulation (*WRKY63, B; MYB29, C*) of glucosinolates and synthesis of a key enzyme of indole-3-acetic acid synthesis (*CYP71A13, D*) to the reference gene *EF1α*. Plants were subjected to 40% PEG 20,000 (severe drought stress) for seven days. Plants were grown on petri dishes for five weeks prior to application of drought stress. Bars represent means of three biological replicates consisting of four pooled plants each. Different letters indicate significant differences (p<0.05) between control and drought-stressed plants.

Supplementary Table S12 List of all mutants analyzed in this study.

Mutant name	TAIR code	Mutant information
nsp1	At3g16400	SALK_072600C
nit2-cluster	At3g44300/<br>At3g44310/<br>At3g44320	RNAi mutant provided by Stephan Pollmann (Lehmann et al., 2017)
bglu18	At1g52400	SALK_075731C
cyp71A13	At2g30770	SALK_105136
cyp79B2/B3	At4g39950/<br>At2g22330	provided by John L. Celenz (Zhao, 2002)
esp	At1g54040	SALK_055029C

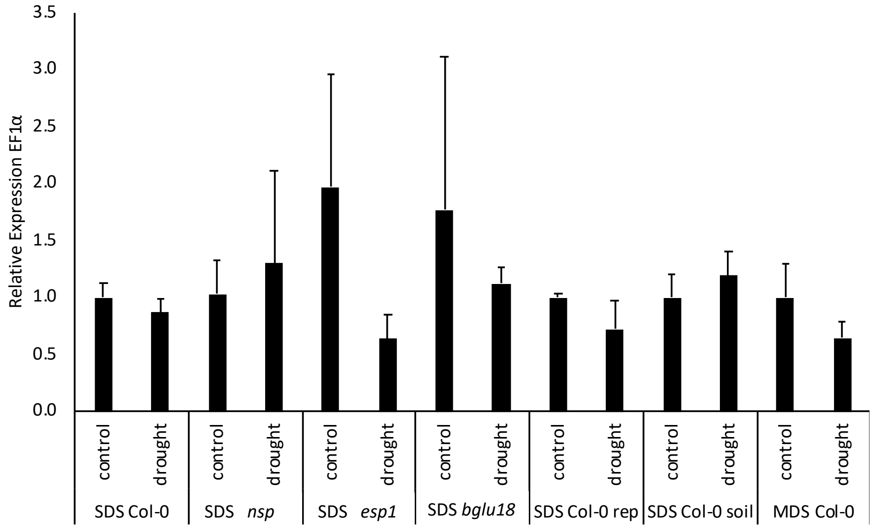

Supplementary Figure S13 Expression of the reference gene *EF1α* relative to the expression in *A. thaliana* Col-0 under standard growing conditions (control).

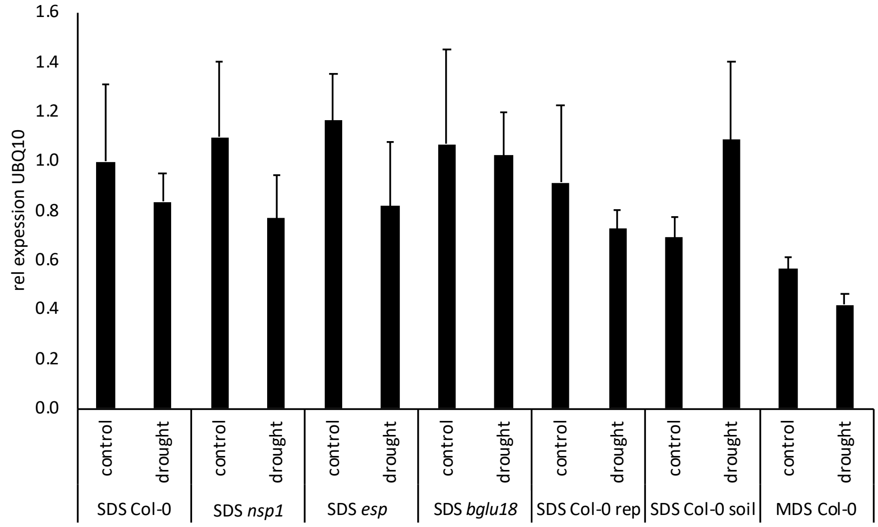

Supplementary Figure S14 Expression of the reference gene *UBQ10* relative to the expression in *A. thaliana* Col-0 under standard growing conditions (control).

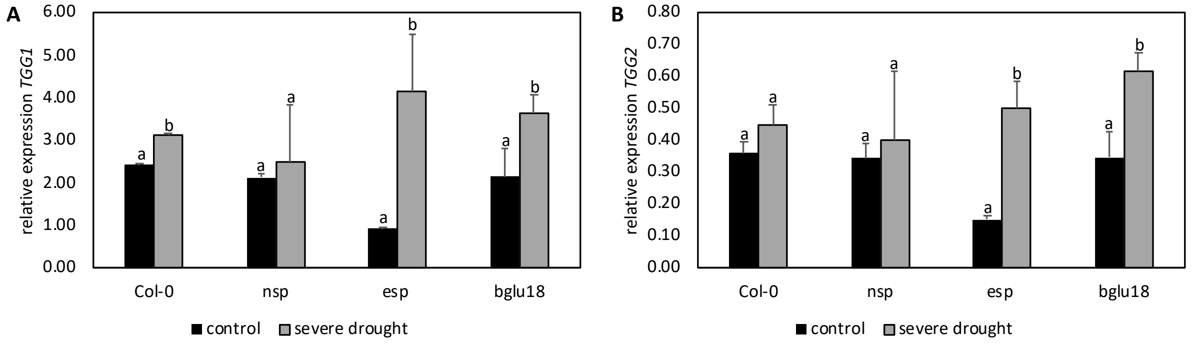

Supplementary Figure S15 Expression of *TGG1* (A) and *TGG2* (B) to the reference gene EF1α. Plants were subjected to 40% PEG 20,000 (severe drought stress) for seven days. Plants were grown on petri dishes for five weeks prior to application of drought stress. Bars represent means of three biological replicates consisting of four pooled plants each. Different letters indicate significant differences ($p<0.05$) between control and drought-stressed plants.

Supplementary Table S16 Mass transitions and optimized parameters for detection of metabolites by mass spectrometry [a].

Metabolite	RT [min]	Ionization mode	Q1 [*m*/*z*]	Q3 [*m*/*z*]	DP [V]	EP [V]	CE [V]	CXP [V]
Raphanusamic acid	0.8	Positive	164	118	42	8	19	4
Indole-3-acetonitrile	3.6	Positive	157	130	90	8	15	8
Indole-3-acetonitrile [b]	3.6	Positive	157	117	90	8	35	8
Indole-3-acetic acid	3	Positive	176	130	31	4	17	4
2-oxothiazolidine-4-carboxylic acid	0.7	Positive	148	102	48	9.5	19	4
D_5-indole-3-acetic acid	3.0	Positive	181	134	41	10	21	4

[a] RT is the retention time. The ionization mode depicts the polarity of the nanoESI source. Q1 and Q3 are the parent and product ion, respectively. CP, EP, CE, and CXP indicate the declustering potential, entrance potential, collision energy, and cell exit potential for each metabolite.
[b] The identity of indole-3-acetonitrile was verified with a second mass transition.

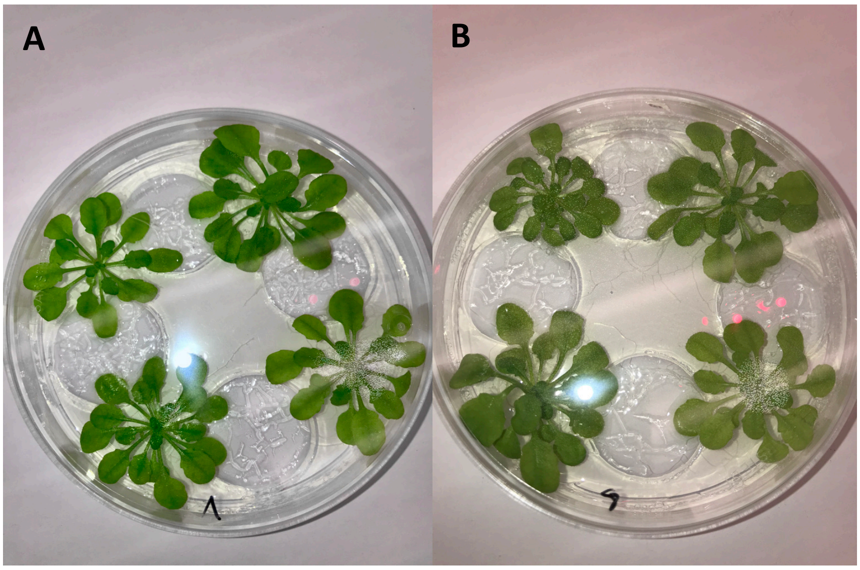

Supplementary Figure S17 Control plants of Col-0 (A) and *cyp79b2/b3* (B). Plants were grown for 6 weeks on solidified ½ MS media.

Supplementary Table S18 Monoisotopic and isotopomeric mass fragments of glucosinolates analyzed as desulfo-glucosinolates.

**Glucosinolate**	**monoisotopic mass fragments** [*m*/*z*]	**Isotopomeric mass fragments** [*m*/*z*]
Glucoiberin	343, 378	379-388
Glucoraphanin	357, 392	393-402
Glucoalyssin	371, 406	407-418
Glucoerucin	341, 376	377-388
Glucohirsutin	413, 448	449-456
Glucobrassicin	367, 402	403-419
4-Methoxyglucobrassicin	398, 433	434-445
Neoglucobrassicin	398, 433	434-458

Supplementary Table S19 Trivial and semi-systematic names of glucosinolates (GSLs) analyzed in this study according to Blažević et al. (2020).

**Trivial name**	**Semi-systematic name**
Glucoraphanin	(R_S)-4-(Methylsulfinyl)butyl GSL
Glucoiberin	(R_S)-3-(Methylsulfinyl)propyl GSL
Glucoalyssin	(R_S)-5-(Methylsulfinyl)pentyl GSL
Glucoarabishirsutain	7-Methylthiohexyl GSL
Glucoerucin	4-(Methylsulfanyl)butyl GSL
Glucobrassicin	Indol-3-ylmethyl GSL
4-Methoxyglucobrassicin	4-Methoxyindol-3-ylmethyl GSL
Neoglucobrassicin	1-Methoxyindol-3-ylmethyl GSL
